# Esculentoside A rescues granulosa cell apoptosis and folliculogenesis in mice with premature ovarian failure

**DOI:** 10.18632/aging.103609

**Published:** 2020-08-05

**Authors:** Zhenteng Liu, Fenghua Li, Jingwen Xue, Meimei Wang, Shoucui Lai, Hongchu Bao, Shunzhi He

**Affiliations:** 1 Department of Reproductive Medicine, Yantai Yuhuangding Hospital, Affiliated Hospital of Qingdao University, Yantai 264000, Shandong, People's Republic of China

**Keywords:** Esculentoside A, premature ovarian failure, granulosa cell

## Abstract

Follicular atresia is one of the main processes for the loss of granulosa cells and oocytes from the mammalian ovary and any impairment to premature ovarian failure. Large numbers of studies have demonstrated that granulosa cell apoptosis causes follicular atresia, yet the rescue of these cells remains elusive. We aimed to use Esculentoside A (3-O-b-D-glucopyranosyl-1, 4-b-D-xylopyranosyl) phytolaccagenin, a saponin extracted from *Phytolacca esculenta* roots, as a potential rescue agent for the apoptosis of granulosa cells. Our results revealed the rescue of normal body and ovary weights, normal ovarian histo-architecture of ovaries, and hormones levels with regular estrus cycle. Consistently, the expression of proliferating and anti-apoptotic markers, i.e. KI67 and BCL-2 in granulosa cells, was enhanced. Meanwhile, the expressions of pro-apoptotic markers, which were BAX and CASPASEs (CASPASE-9 and CASPASE-3), were prominently reduced in Esculentoside A-induced premature ovarian failure mice. Additionally, PPARγ, a potential therapeutic target, has also rescued its expression by treating the premature ovarian failure mice with Esculentoside A. Our results advocated that Esculentoside A could restore folliculogenesis in premature ovarian failure mice. Furthermore, it has the potential to be investigated as a therapeutic agent for premature ovarian failure.

## INTRODUCTION

A mammalian ovary ovulates less than 1% of follicles, and the rest follicles undergo atresia in various phases of growth [1]. Therefore, follicular atresia is considered as one of the major processes leading to the loss of follicles and oocytes from the ovary, and any impaired follicular atresia lead to premature ovarian failure (POF) [2]. Previous reports elaborated that the cellular mechanisms responsible for follicular atresia would lead to the apoptosis of granulosa cells (GCs) [3]. GC apoptosis is caused by a large number of factors including hormones, reactive oxygen species (ROS), growth factors, cytokines, and *Bcl-2* family members [4], yet its underlying molecular mechanisms have not been described fully.

GCs lie outside the zona pellucida in an individual follicle and play essential roles in folliculogenesis [5, 6]. They nourish and regulate the oocyte development by establishing physical connections known as gap junctions [6]. GCs can also synthesize and release a variety of growth factors and hormones that can regulate the growth, differentiation, and maturation of oocytes and theca cells. Among these factors, the most important steroids are estrogens. The androgens secreted by the theca cells are converted by activated aromatase in GCs into estrogens after a series of biochemical reactions. Aromatase, like other enzymes involved in the steroid hormone biosynthesis pathway, is located in the membrane of endoplasmic reticulum. Follicle stimulating hormone (FSH) enhances the expression and activity of aromatase in GCs of several mammalian species, including rodents and humans [7–9]. Notably, follicle stimulating hormone receptor (FSHR) on GCs first appears at the preantral stage of folliculogenesis, which indicates their dependence on FSH. Hence, GCs from preantral and onward follicles produce estrogens, which play vital roles in follicle development and oocyte maturation [10–12]. It is well-established that GCs act as the main sources of estrogen and progesterone hormones [4, 13, 14]. The presence of GCs is indispensable for oocyte development as it can in large part meet the needs of oocyte metabolism (such as amino acids, ions, and metabolites, etc.) via gap junctions [15, 16]. In addition, GCs also regulate the activity of oocyte at transcriptional level and facilitate the post-transcriptional modifications of a number of oocyte proteins [17]. Hence, the normal functions of GCs are of prime importance for folliculogenesis and any factors that may lead to POF. Therefore, the rescue of GCs would be one of the potential targets to prevent the development of POF.

Esculentoside A (EA) is a saponin extracted from *Phytolacca esculenta* roots and has been recognized as 3-O-[b-D-glucopyranosyl-(1,4)-b-D-xylopyranosyl] phytolaccagenin [18]. It is well known that EA can impede the secretion of inflammatory mediators such as tumor necrosis factor (TNF)-α, interleukin (IL)-1β, IL-6, and prostaglandin E-2. Reports have also revealed that EA has the potential to reduce the over-activated macrophages by modulating phagocytosis and inhibiting the secretion of inflammatory cytokines [19, 20]. Similarly, studies have shown that EA can lessen the severity of both early and late radiation-induced cutaneous toxicity [21]. Z. Wei-ting., et al elaborated that EA can inhibit nuclear factor kappa B and protein kinase signaling pathways in lipopolysaccharide (LPS)-induced acute lung injury (ALI) mice, resulting in the reduction of inflammatory responses [22]. Similarly, EA has also been shown to inhibit the ectopic lesions growth in an Endometriosis (EMS) animal model (China Patent Number: CN200510110906.X). It is obvious that EA has great potential to rescue various abnormal conditions. Hence, the therapeutic potential of the EA urged us to study whether it could be used as revival agent for POF.

This study aimed to investigate the restoration of GCs in POF mice via EA administration. POF mice were selected and divided into two groups: POF group and EA-administered POF group. EA-administered POF mice were further classified into three groups depending upon the doses: 15, 30, 60 mg/day for 4 weeks. The successive examinations showed that the attained body and ovary weight, ovarian histology, estrus cycle and hormones of POF mice (given EA with dose of 60 mg/day) were equivalent to the control of the same age. Similarly, the expression of proliferating and anti-apoptotic markers in GCs was enhanced, and the expression of pro-apoptotic markers was significantly decreased in EA-administered POF mice when compared with the POF mice. Furthermore, PPARγ, a potential therapeutic target, has similar expression profile in POF mice treated with the highest dose of EA compared to that in control group. All these results suggested the restoration of GC activities and folliculogenesis.

## RESULTS

### Body and ovary weights in POF mice

**Figure 1:**
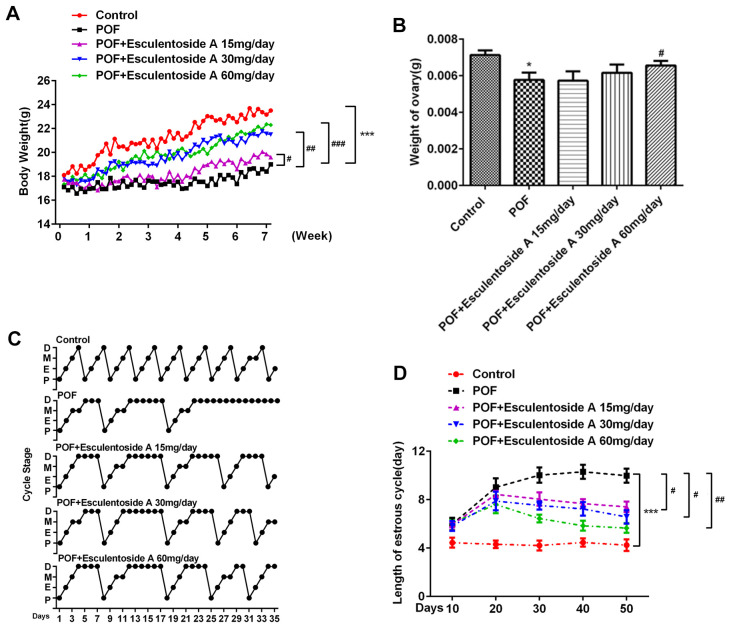
**Effect of Esculentoside A on body, ovary weights and estrus cycle of POF mice.** (**A**, **B**) Body weight and ovary weight after combination of POF and EA treatment, respectively. POF: Premature ovarian failure (**C**) Representative picture of estrous cycles in control mice (upper), POF mice (middle) and Esculentoside A-treated mice (lower). Bars represent the average length (day) of proestrus (P), estrus (**E**), metestrus (M) and diestrus (**D**) per estrous cycle. (**D**) The length of estrous cycle of each group. ^*^P< 0.05, ^***^P < 0.001 *vs.* Control. *^#^P* < 0.05, ^##^P < 0.01, *^###^P* < 0.001 *vs.* POF.

The body weight of POF mice was recorded as significantly reduced when compared with normal control of the same age (Figure 1A). To check the effects of EA and body weights of POF mice, we further divided them into 4 groups based on different doses given, i.e., null EA (POF mice to which EA was not administered), 15, 30 and 60 mg/day. The treatment was carried out for 28 consecutive days and the weight for each group was recorded accordingly. The results revealed that the weight of POF mice was directly proportional to the doses given. The POF mice with the highest dose of EA (60mg/day) had recovered the weight analogous to the normal control. In detail, the weight of 6-week-old POF mice was 22.3 grams (g), bordering on that of normal control. In contrast, the weight of null EA POF mice of the same age reached about 19g. Similarly, a positive correlation between the dose of EA and ovarian weight in POF mice was also observed (Figure 1B). In detail, the utmost ovary weight was recorded in POF mice with the highest dose of EA as compared with the null EA POF mice with the lowest dose. Taken together, these results suggested that EA treatment restored both normal body and ovary weights in POF mice.

### Estrogenic activity in POF mice

Next, we investigated whether EA could restore the estrus cycle in POF mice (as the cycle was found absent from these mice). The POF mice were divided into 4 groups as mentioned earlier. Vaginal smears were obtained from all groups including normal control to identify the different phases of estrus cycle (Figure 1C, 1D). In comparison with EA-null POF mice, the EA-administered POF mice restored their estrus activity to the level close to normal control. Furthermore, EA also provoked vaginal opening, which was found to be directly proportional to the EA dose. Hence, the mice administered with the highest dose of EA (60mg/day) demonstrated vaginal opening comparable to normal control. These observations determined that EA successfully rescued the estrus cycle in POF mice.

**Figure 2:**
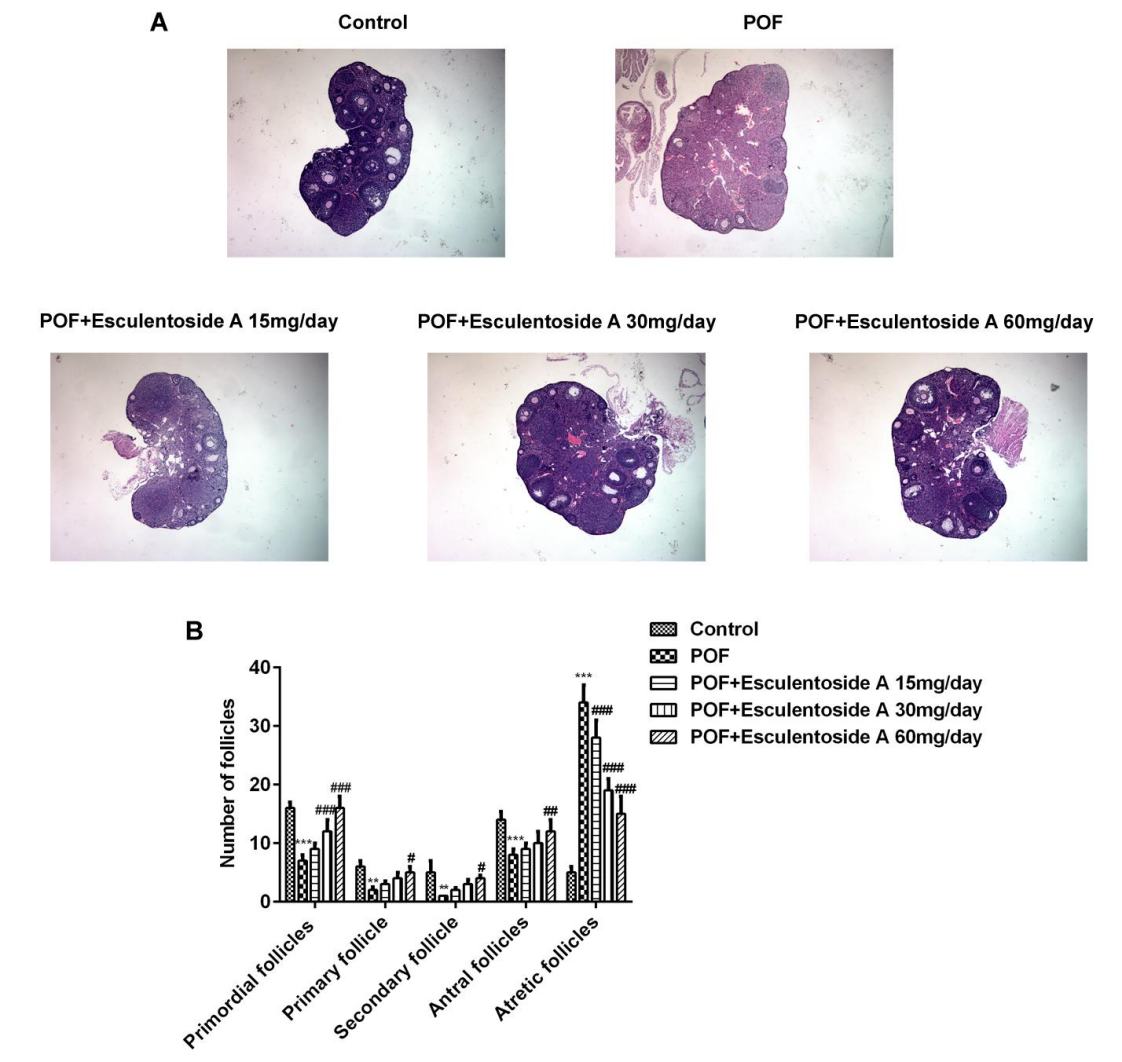
**Effect of Esculentoside A (EA) on follicle growth and ovulation in POF mice.** (**A**) Representative picture of ovaries stained by H&E. (×400 magnification). (**B**) The mean number of primordial follicles, primary follicles, secondary follicles, antral follicles and atretic follicles. The data were expressed as means ± SD (n =5). ^***^P < 0.001 *vs.* Control. *^#^P* < 0.05, ^##^P < 0.01, *^###^P* < 0.001 *vs.* POF.

The estrus cycle restoration in POF mice by EA urged us to evaluate the histopathological conditions of their ovaries. Subsequently, histological analysis of POF mice ovaries revealed typically atretic follicles, which were characterized by disintegration of granulosa and theca cell layers. In addition, a number of granulosa cells with pyknotic nuclei were detected in the atretic follicles, and cumulus oophorus disappeared. Moreover, aged oocytes with germinal vesicle breakdown (GVBD) were observed. In contrast, ovarian morphology after EA treatment showed the normal appearance of growing antral follicles, and decreasing propensities for atresia compared with the POF mice. It was observed that in growing antral follicles, granulosa and theca cell layers maintained their normal integrities and the oocytes without GVBD appeared similar to normal control (Figure 2). In short, compared with POF mice, the number of atretic follicles was decreased, whereas the number of the other follicles, including primordial follicles, primary follicles, secondary follicles and antral follicles, was increased.

### Hormone levels in POF mice

**Figure 3:**
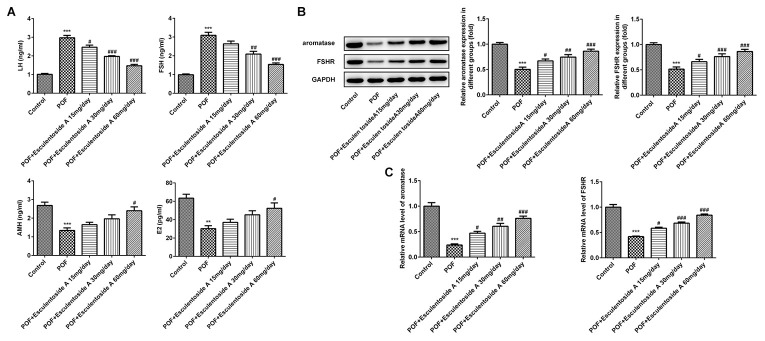
**Effect of Esculentoside A (EA) on reproductive hormone levels in POF mice.** (**A**) The levels of serum luteinizing hormone (LH), FSH, anti-mullerian hormone (AMH) and estradiol (E2) from normal control, POF (EA-treated and EA-null) were detected by ELISA kits. (**B**–**C**) The levels of aromatase and FSHR of proteins and mRNA were evaluated by western blot and RT-qPCR, respectively. The data were expressed as means ± SD (n =5). ^**^P < 0.001, ^***^P < 0.001 *vs.* Control. *^#^P* < 0.05, ^##^P < 0.01, *^###^P* < 0.001 *vs.* POF.

EA treatment prompted estrus cycle and follicular activity in POF mice and it has been well established that both of them are regulated and maintained by certain endocrine hormones, i.e., luteinizing hormone (LH), FSH, anti-mullerian hormone (AMH) and estradiol. To check the levels of these hormones in POF mice, we performed enzyme-linked immunosorbent (ELISA) assay on serum collected from normal control, EA-null POF mice, and EA-administered POF mice (with dose of 15, 30 and 60 mg/day). Our results demonstrated that EA-null POF mice had abnormally high levels of LH and FSH, and low levels of AMH and E2. By contrast, with increasing doses of EA, the hormone level of POF mice was also corrected, while the hormone level of POF mice with 60 mg/day EA bordered on that of normal control (Figure 3A). To further validate the activity of FSH, we assessed the expression of FSHR and aromatase in ovarian tissue by qRT-PCR and WB. The results showed that they exhibited comparable mRNA and protein expressions (Figure 3B, 3C). Down-regulated expressions of aromatase and FSHR were observed in POF mice, which can be reversed by EA in a dose-dependent manner. Hence, consistent with the aforesaid observations, EA also reinstated the levels of hormones, FSHR and aromatase.

### Granulosa cells proliferation in POF mice

**Figure 4:**
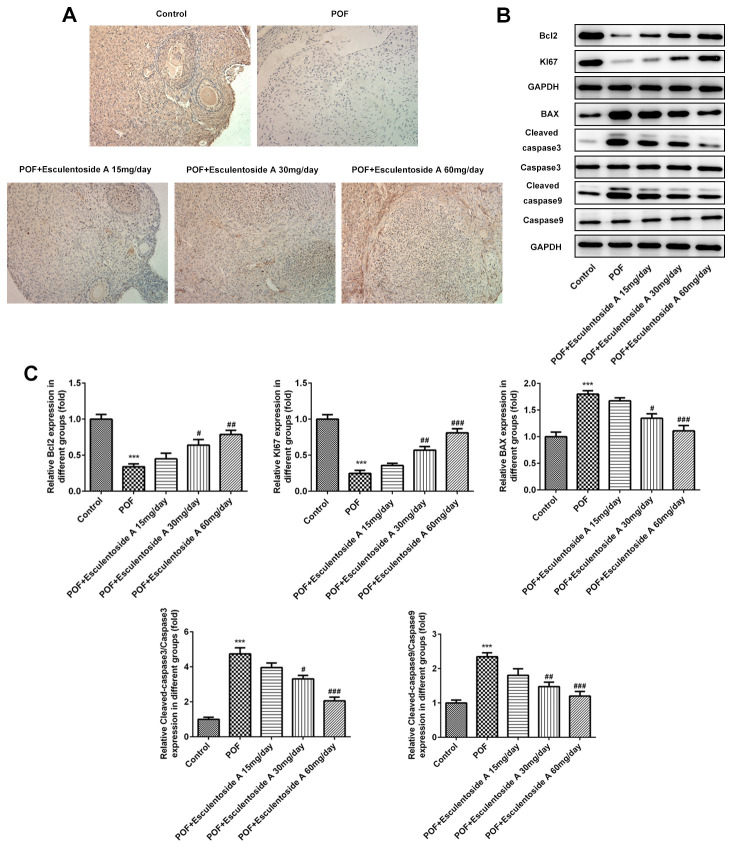
**Effect of Esculentoside A (EA) on proliferation and apoptosis of ovarian granulosa cells.** (**A**) The expression of KI-67 of granulosa cells was determined by immunohistochemistry of ovarian tissue. KI-67 was used as positive marker of cell proliferation. (**B**, **C**) The expressions of proteins including BCL2, KI-67, BAX, CASPASE 3 and CASPASE9 were detected by western blot. The data were expressed as means ± SD (n =5). ^***^P < 0.001 *vs.* Control. *^#^P* < 0.05, ^##^P < 0.01, *^###^P* < 0.001 *vs.* POF.

**Figure 5:**
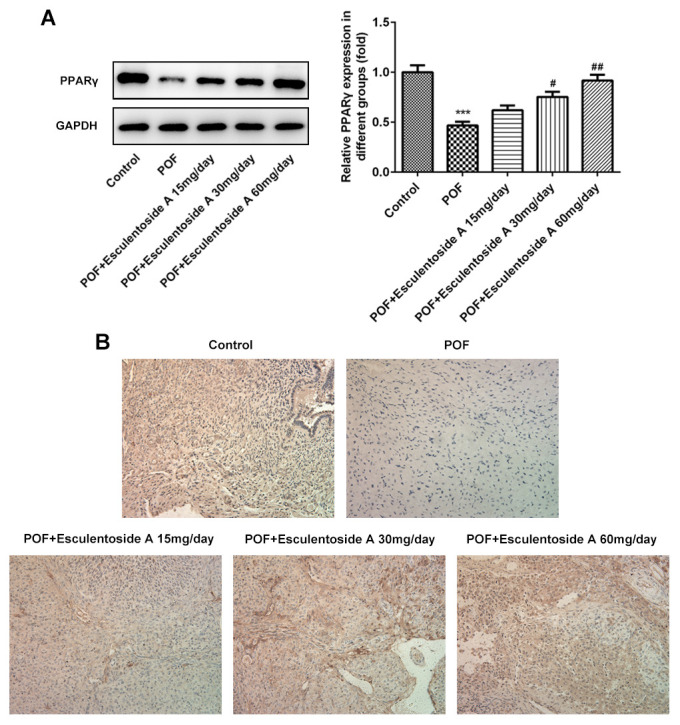
**Effect of Esculentoside A (EA) on PPARγ signaling protein expression.** (**A**) The expression of PPARγ proteins was detected by western blot. (**B**) The expression of PPARγ of ovarian tissue was determined by immunohistochemistry. The data were expressed as means ± SD (n =5). ^***^P < 0.001 *vs.* Control. *^#^P* < 0.05, ^##^P < 0.01 *vs.* POF.

The GCs convert androgens into estrogens and synthesize progesterone, thus playing essential roles in the oogenesis and ovary maintenance [5]. Based on earlier results, it was hypothesized that whether the GCs were retained in the ovaries from POF mice. To check the status of GCs, we carried out immunohistochemistry (IHC) on ovaries from normal control, EA-null POF mice and EA-treated POF mice using KI-67 (GCs proliferating marker). We could not determine specific KI-67 signals in GCs from the ovaries of EA-null POF mice, compared with normal control. In contrast, the EA-treated mice showed KI-67 signals and the strengths of signals were illustrated by the EA dose given. Consistently, the POF mice to which the highest EA dose was given exhibited KI-67 signals similar to normal control (Figure 4A). To further validate the proliferation of GCs, we checked the expression of KI-67 along with another anti-apoptotic marker, i.e., BCL-2 and pro-apoptotic makers: BAX and CASPASEs (CASPASE-9 and CASPASE-3) using western blot analysis (Figure 4B, 4C). Our result showed the reversed expression of these markers in EA-treated POF mice as compared with EA-null POF mice. The expression of KI-67 and BCL-2 in EA-treated POF mice with the highest dose was equivalent to that of normal control. Similarly, the decreased expression was observed to the levels reported in the normal control for BAX and CASPASEs. Moreover, PPARγ can be considered as a potential therapeutic target as it impedes proliferation and promotes terminal differentiation of GCs [23]. Therefore, we investigated the expression of PPARγ in GCs of EA-treated POF mice by western blot (WB) and IHC (Figure 5A, 5B). The result from WB and IHC demonstrated similar expression profile of PPARγ in POF mice administered with the highest EA dose. Taken together, these results were consistent and EA treatment redirected the GC proliferation and its normal functions.

## DISCUSSION

In this study, we for the first time demonstrated that EA administration restored folliculogenesis and GCs in POF mice. Briefly, EA with doses of 15, 30, 60 mg/day were given for 28 days to these mice. The re-establishment of normal body and ovary weights, ovarian histology, estrus cycle and normal hormones levels were observed in aforementioned groups. Consistently, the enriched and reduced expressions of proliferating markers (KI-67 and BCL-2) and pro-apoptotic markers (BAX and CASPASES) in GCs were determined respectively. In addition, EA administration also prompted a potential therapeutic target: PPARγ with expression profile in POF mice equivalent to normal control.

EA is a perennial herb derivative mainly extracted from *Phytolacca esculenta*. Many studies provided enough evidence with respect to the physiological importance of EA. The reports elaborated that EA plays a vital role in regulating immune response [24], cell proliferation [25] and apoptosis [26]. EA has also possessed anti-inflammatory properties, and protective effects on liver toxicity and lung damage [22, 27]. Similarly, Chuanlan et al., demonstrated EA inhibits breast cancer stem cell (CSC) growth both in vitro and in vivo [28]. Therefore, we investigated whether EA could be a promising candidate for the treatment and prevention of POF. The consequent examinations using POF mice illustrated that EA has offered the great potential to be used as a rescue agent for POF treatment for woman.

POF is an abnormal condition in which the ovarian follicles rapidly decrease with no or few residual follicles in women aged fewer than 40 years [29, 30]. The other features of this multifactorial disease include hypo-estrogenism, elevated levels of gonadotrophin, amenorrhea, and infertility [31]. A variety of factors have been found to be associated with POF including genetic defects, infections, autoimmunity, drugs, and toxics [32]. Approximately 10% of worldwide women of age between 30 and 39 are suffering from POF [30]. The heterogeneity and promising percentage of POF offer great opportunities for the proper treatment of this disease condition. In one such effort, Zhengjie et al., proved that curcumin is a protective agent against POF in mice [33]. Interestingly, in this study, we for the first time determined that EA could completely rescue the POF in the animal model, therefore it would be more fascinating to check whether EA has similar effects on human.

Estrogens and progesterone are responsible for the regulation of histological and functional modifications of female genital tract. It has also been well established that the estrous is primarily regulated by estrogen, which, in turn, is monitored by hypothalamic-releasing factors (HRF) and pituitary gonadotropins [34]. Consistent with previous reports, in this study, we demonstrated that the exogenous administration with physiological doses of EA in POF mice restored the complete phenomena of sexually and reproductively active mice, i.e.. normal histo-architecture of ovaries, estrus cycle, and normal levels of hormones, suggesting the restoration of normal GC activities along with oocyte development.

At molecular levels, GC apoptosis follows two main pathways: death receptor and mitochondrial pathways [35]. In the earlier pathway, the specific death ligands will combine with death receptors, thus activating caspase cascade [36]. In the mitochondrial pathway, the pro-apoptotic signals such as *Bax* will initiate the apoptosis and promote follicular atresia [37]. In the process of cell apoptosis, BAX moves to mitochondria from cytoplasm, which subsequently enhances the mitochondrial membrane permeability. As a result, cytochrome C is removed from mitochondria to cytoplasm where it makes a complex with apoptotic protease-activating factor-1 (*Apaf-1*). By the successive mechanism, this complex activates *Caspase-9* and *Caspase-3* that promote cell apoptosis. BCL-2 prohibits the cytochrome C elimination from entering into the cytoplasm, thus acting antagonistically on BAX and opposing cell apoptosis [38–40]. Therefore, the expression ratio of *Bcl-2* to *Bax* and its successors shows the ‘molecule switch’ of apoptosis [41, 42]. Furthermore, we have consistent findings on the underlying mechanism of POF. The POF mice revealed higher expression of BAX, CASPASE-9 and CASPASE-3 as compared with BCL-2, whereas the reversed ratio was observed in EA-administered POF mice, confirming the proliferation of GCs and restoration of folliculogenesis. Moreover, we have observed that the expression of a potential therapeutic receptor, PPARγ, which was found to be high expressed in EA-administered POF mice. A number of reports illustrated the apoptotic property of PPARγ [43, 44]. Compared with these studies, Lefebvre et al., and Saez et al., independently demonstrated that activation of PPARγ promoted cell proliferation in colon tumors [45, 46]. Our results are consistent with the previous findings and strongly suggested that PPARγ plays an essential role in cell maintenance and division. Furthermore, the increased expression of PPARγ and proliferation- and apoptosis-related proteins indicates the same molecular pathway for them. But the mechanism by which this pathway works offers us with some valuable investigations.

In conclusion, the present study highlights the potential of EA to restore reproductive ability with all its basic features in an infertile female mouse suffering from POF. We found which the expression of anti-apoptotic protein BCL-2 was elevated, and BCL-2 prohibits the GCs from apoptosis via mitochondrial pathway in the POF mice. Moreover, EA provoked the expression of the potential therapeutic target, PPARγ, in POF mice. It is highly appreciated, if the EA consequences would be investigated in infertile woman suffering from POF with GC atresia.

## MATERIALS AND METHODS

### Mice and treatment

Mice purchased from Oriental Bio Service Inc. (Nanjing) were kept in the animal house. The temperature and humidity were maintained between 23°C-25°C and 40%-80% respectively. The animal-involved experiments were performed according to the guidelines provided for the Care and Use of Laboratory Animals published by the US National Institutes of Health and consequently approved by the Institutional Ethics Committee of Yantai Yuhuangding Hospital and Affiliated Hospital of Qingdao University.

The mice were divided into the Control group (n = 5), the POF group (n = 5) and three POF+Esculentoside A groups (n = 5). The Control group received the same weight-based volume saline only. The POF group and the POF+Esculentoside A group were injected intraperitoneally with 75 mg/kg cyclophosphamide (CY, Aladdin). The POF+Esculentoside A groups were administered intragastrically at the dosage of 15, 30, 60 mg/day for four weeks.

### Chemical reagents

EA (purity, >92.2%) was purchased from Nanjing Spring and Autumn Biological Engineering Co., Ltd., Nanjing, China. ELISA kit was obtained from Uscn Life Sciences, Inc., Wuhan, China. The antibodies against KI67, BCL-2, BAX, CASPASE-9, CASPASE-3 and PPARγ were purchased from Millipore, Billerica, MA, USA.

### RNA extraction and qualitative real-time polymerase chain reaction (qRT-PCR)

Control groups and treated groups’ total RNAs were extracted by the TRIzol Reagent Kit (Invitrogen, Grand Island, NY, USA) according to the given protocol. RevertAid™ First Strand cDNA Synthesis Kit (Fermentas MBI, Waltham, MA, USA) was used for reverse transcription. qRT-PCR was carried out with SYBR Green PCR Master Mix (Thermo Scientific, Waltham, MA, USA). iCycler IQ Multicolor Detection System (Bio-Rad, Hercules, CA, USA) was used to run the PCR cycles. The PCR reactions were carried out under the following optimized conditions: 15 minutes (min) at 95°C, followed by 40 cycles of 15 sec at 95°C, and 30 sec at 72°C. Fold change in gene expression was determined by the comparative cycle threshold (CT) method. The sequences of all primers were as below:

**Table:** 

Aromatase	Forward	5’- ATGTTCTTGGAAATGCTGAACCC - 3’
	Reverse	5’- AGGACCTGGTATTGAAGACGAG - 3’
FSHR	Forward	5’- AGCCCCTTGTCACAACTCTATGTC - 3’
	Reverse	5’- AGCCCCTTGTCACAACTCTATGTC - 3’
GAPDH	Forward	5’-CTCACCGGATGCACCAATGTT- 3’
	Reverse	5’-CGCGTTGCTCACAATGTTCAT- 3’

### Western blot assay

To obtain the lysates, ovaries were washed with ice-cold phosphate buffer saline (PBS) and lysed in 1× sodium dodecyl sulfate (SDS) buffer (100 mM Tris-HCl pH 7.4, 2% SDS, 15% glycerol, 0.1% bromophenol blue and 5 mM dithiothreitol). For western blot, the specific primary antibodies against anti-KI-67, anti-BCL-2, anti-BAX, anti-CASPASE-9 and anti-CASPASE-3 (Santa Cruz Biotechnology, Santa Cruz, CA, USA) were diluted at 1:1000 and the previously described procedure was followed [47].

### Hematoxylin and Eosin (HE) staining

The mice (70-day-old) were euthanized by cervical dislocation. For fixation, after the removal, ovaries were instantly put in Bouin’s solution and left overnight at room temperature. Paraffin-embedded ovaries were sectioned by microtome and subsequent HE staining was carried out as described previously [48]. The same staining procedure was applied for vaginal smear to assess the estrus cycle. Digital Nikon DS-Ri1 camera installed on a Nikon Eclipse 80i microscope was used to capture image.

The follicular (primordial, primary, secondary, early antral, antral, and preovulatory follicles) count was carried out in every six sections (30 lm apart). Subsequently, to get the total number of follicles per ovary, each section was multiplied by 6. Importantly, to avoid any doubling, oocytes with visible nucleus were considered for counting. Myers et al*.’*s morphological criteria were followed for the classification of follicular stages [49]. Glidewell-Kenney et al. described that the procedure was used for the count of corpora lutea, that is to say, 1 section per ovary and 1 ovary per mouse [50].

### Immunohistochemical staining

Fixation of the ovaries was performed for 20 minutes in 4% paraformaldehyde (PFA) at room temperature, followed by permeabilization in 0.2% Triton X-100 supplemented phosphate puffer saline (PBS) for 15 minutes. Ovaries were incubated with primary antibodies at 37°C for 2 hours later to block them with 5% normal donkey serum (Jackson ImmunoResearch, 017-000-121). Then, PBS was added for 30 minutes. Afterwards, secondary antibodies were added for 1 hour at room temperature. Ovaries were shifted to slides later to be stained with Hoechst 33342 (Invitrogen, H21492). The slides were mounted by Vectashield (Vector Laboratories, H-1000). Fluorescence was detected on an Eclipse 80i microscope (Nikon) equipped with a digital camera (Hamamatsu, C4742-80).

### Statistical analysis

The data are expressed as mean ± SEM. Each experiment was performed in triplicate. All statistical analyses were performed with the SPSS 19.0 using one-way analysis of variance (ANOVA) test followed by Bonferroni’s post hoc test among multiple groups. P < 0.05 was considered statistically significant.
